# NirA Is an Alternative Nitrite Reductase from Pseudomonas aeruginosa with Potential as an Antivirulence Target

**DOI:** 10.1128/mBio.00207-21

**Published:** 2021-04-20

**Authors:** Samuel Fenn, Jean-Frédéric Dubern, Cristina Cigana, Maura De Simone, James Lazenby, Mario Juhas, Stephan Schwager, Irene Bianconi, Gerd Döring, Jonas Elmsley, Leo Eberl, Paul Williams, Alessandra Bragonzi, Miguel Cámara

**Affiliations:** a National Biofilms Innovation Centre, Nottingham University Biodiscovery Institute, School of Life Sciences, University of Nottingham, Nottingham, United Kingdom; b Division of Immunology, Transplantation and Infectious Diseases, IRCCS San Raffaele Scientific Institute, Milan, Italy; c Department of Microbiology, Institute of Plant Biology, University of Zürich, Zürich, Switzerland; d Institute of Medical Microbiology and Hygiene, University of Tübingen, Tübingen, Germany; Emory University School of Medicine

**Keywords:** *Pseudomonas aeruginosa*, virulence target, nitrite reductase, transposon mutagenesis, genome-wide screening, disease model, disease models, virulence determinants

## Abstract

The opportunistic pathogen Pseudomonas aeruginosa produces an arsenal of virulence factors causing a wide range of diseases in multiple hosts and is difficult to eradicate due to its intrinsic resistance to antibiotics. With the antibacterial pipeline drying up, antivirulence therapy has become an attractive alternative strategy to the traditional use of antibiotics to treat P. aeruginosa infections. To identify P. aeruginosa genes required for virulence in multiple hosts, a random library of Tn*5* mutants in strain PAO1-L was previously screened *in vitro* for those showing pleiotropic effects in the production of virulence phenotypes. Using this strategy, we identified a Tn*5* mutant with an insertion in PA4130 showing reduced levels of a number of virulence traits *in vitro*. Construction of an isogenic mutant in this gene presented results similar to those for the Tn*5* mutant. Furthermore, the PA4130 isogenic mutant showed substantial attenuation in disease models of Drosophila melanogaster and Caenorhabditis elegans as well as reduced toxicity in human cell lines. Mice infected with this mutant demonstrated an 80% increased survival rate in acute and agar bead lung infection models. PA4130 codes for a protein with homology to nitrite and sulfite reductases. Overexpression of PA4130 in the presence of the siroheme synthase CysG enabled its purification as a soluble protein. Methyl viologen oxidation assays with purified PA4130 showed that this enzyme is a nitrite reductase operating in a ferredoxin-dependent manner. The preference for nitrite and production of ammonium revealed that PA4130 is an ammonia:ferredoxin nitrite reductase and hence was named NirA.

## Abstract (precis)

The emergence of widespread antimicrobial resistance has led to the need for development of novel therapeutic interventions. Antivirulence strategies are an attractive alternative to classic antimicrobial therapy; however, they require identification of new specific targets which can be exploited in drug discovery programs.

## INTRODUCTION

Pseudomonas aeruginosa is a genetically versatile opportunistic pathogen, able to colonize and survive in multiple environments and hosts. This versatility underpins the ability of P. aeruginosa to cause a wide range of infections, commonly affecting the respiratory tract, burn wounds, urinary tract, bloodstream, cornea, skin, and soft tissue (1). The majority of these infections are nosocomial, with infections in immunocompromised hosts often life-threatening.

P. aeruginosa has gained notoriety as a member of the ESKAPE (Enterococcus faecium, Staphylococcus aureus, Klebsiella pneumoniae, Acinetobacter baumannii, Pseudomonas aeruginosa, and *Enterobacter* species) pathogens (2). These pathogens are differentiated according to their clinical relevance and capacity to become multidrug resistant (MDR). Often treatment of P. aeruginosa is unsuccessful due to high levels of intrinsic and acquired antimicrobial resistance with biofilm formation promoting antimicrobial tolerance (3, 4).

Although carbapenem-resistant P. aeruginosa has been listed as “priority one” by the World Health Organization for the development of new antimicrobials, no new drugs with a novel mechanism of action against this organism have reached the market in recent years (5). Hence, there is a pressing need for the discovery of novel alternative strategies to the traditional use of antibiotics to treat P. aeruginosa infections. Antivirulence therapeutic approaches offer an attractive alternative strategy for developing drugs with high specificity and narrow spectra as they reduce the illness caused by the pathogen (“pathogen limitation”) instead of reducing pathogen burden directly (“pathogen elimination”) (6).

In recent years, vast progress has been made on the identification of P. aeruginosa virulence factors, unravelling the mechanisms they employ to cause disease and developing inhibitors which can inactivate them (7–12). While these studies have uncovered numerous promising small virulence inhibitor molecules, none has yet made it to the clinic. This has been influenced by many different factors, including the reliance on a single disease model, potential target conservation within the microbiota, a lack of understanding of target functionality, and the inability to define success when searching for inhibitors (13).

The sequencing of the first P. aeruginosa genome in 2000 revealed that the PAO1 strain sequenced (PAO1-UW) has a genome size of 6.3 Mbp and contains 5,570 open reading frames, making it the largest bacterial genome sequenced at the time (14). This large genome underpins the extensive metabolic and regulatory network providing P. aeruginosa with the genetic versatility to colonize multiple environments, hosts, and host sites. Besides, with the function of only 22.7% of P. aeruginosa genes experimentally demonstrated and close to 2,000 genes without functional annotation (15), there is still a large amount of information missing with regard to the mechanisms by which this organism causes disease, and potentially a vast array of novel P. aeruginosa virulence targets remains to be discovered.

Early studies suggested that virulence mechanisms employed by P. aeruginosa to infect phylogenetically diverse hosts are remarkably well conserved. Comparison of infection mechanisms in the plant Arabidopsis thaliana and mice revealed that P. aeruginosa uses a shared subset of virulence genes to provoke disease (16). The conserved nature of P. aeruginosa virulence suggested that use of a single disease model is sufficient to dissect P. aeruginosa virulence in all hosts (17), with various studies utilizing the nematode Caenorhabditis elegans (18), fruit fly Drosophila melanogaster (19), silkworm Bombyx mori (20), Galleria mellonella larvae (21), and zebrafish embryos (22). However, limitations of these studies are related to the impact of virulence in multihost system and the number of P. aeruginosa strains tested.

A study performed by our group combining whole-genome transposon mutagenesis with a cascade of *in vitro* and *in vivo* infection models uncovered the host-specific nature of P. aeruginosa virulence (23). This revealed a remarkably low overlap in virulence factor requirements between different models, suggesting that many of the virulence factors identified with single model studies may not represent virulence factors required during human disease (23). Given the broad range of clinical manifestations exhibited by P. aeruginosa infections, it stands to reason that virulence genes identified as attenuated in multiple models are more likely to be both relevant to human disease and at multiple infection sites, representing promising antivirulence targets.

This study builds upon the successful whole-genome transposon mutant screening to identify novel P. aeruginosa virulence target genes (23). Here, we describe the identification of an additional mutant from this library on a yet uncharacterized gene of P. aeruginosa, PA4130, exhibiting attenuation in all infection models tested. Functional characterization revealed that PA4130 encodes an assimilatory nitrite reductase with a potential role in nitrogen source metabolism during P. aeruginosa pathogenesis. The attenuation in all disease models tested and the lack of human homologues make this nitrite reductase a promising antivirulence therapeutic target.

## RESULTS

### Isolation of an P. aeruginosa mutant attenuated in multiple virulence factor production.

**FIG 1:**
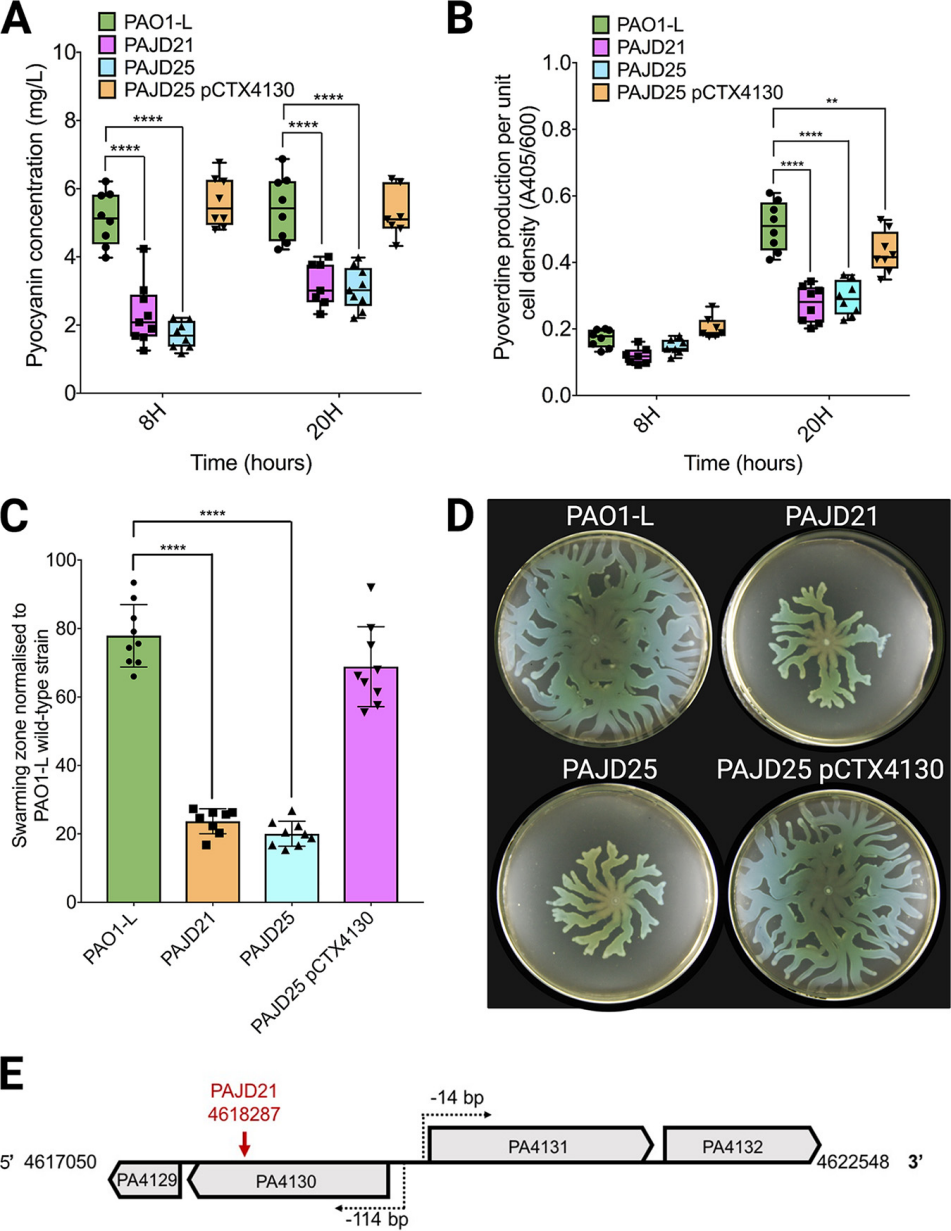
*In vitro* phenotypic characterization of P. aeruginosa PAO1-L virulence factor production versus PA4130 mutants. The transposon mutant PAJD21 (PA4130::Tn*5*), the PA4130 in-frame deletion mutant PAJD25, and the complemented mutant PAJD25 pCTX4130 were used in these experiments. (A) Pyocyanin levels determined in supernatants from cultures grown for 8 and 20 h in LB using a colorimetric assay. (B) Pyoverdine levels determined in supernatants from cultures grown for 8 and 24 h in modified artificial sputum medium using a colorimetric assay. A405/600, absorbance at 405 and 600 nm. (C and D) Swarming motility surface coverage measurements (C) and images (D); cells were inoculated from a 16-h LB culture onto swarming plates containing 0.5% agar and incubated at 37°C for 16 h. (E) Diagram displaying operon structure and transcriptional start sites of PA4129- PA4130 and PA4131- PA4132 with the PAJD21 Tn*5* insertion site indicated by the red arrow. Data were collated from three independent experiments with at least three replicates each. Values that are significantly different by Dunnett’s multiple-comparison test are indicated by asterisks as follows: **, *P* < 0.01; ****, *P* < 0.0001.

To isolate novel virulence genes, a transposon (Tn*5*) insertion library was generated in a wild-type PAO1-L strain (23). A total of 57,360 individual colonies were picked and screened for pleiotropic attenuation in virulence phenotypes (reduced swarming and exoprotease and pyocyanin production) (23). Using the same screening approach, in the current study, a further transposon mutant (PAJD21) was isolated from the library. This mutant displayed reduced levels of pyocyanin and pyoverdine production as well as decreased swarming motility, with twitching, swimming, protease, and elastase activity being unaffected compared to that of the wild type (Fig. 1A to D; see also Table S1 in the supplemental material). Nucleotide sequence analysis of the Tn*5*-flanking region showed that the transposon had inserted into PA4130, encoding a hypothetical protein with homology to nitrite and sulfite reductases, and forming a predicted operon with PA4129 (Fig. 1E). To ensure the attenuation in virulence traits observed was not due a polar effect on PA4129, located in the same predicted transcriptional unit as PA4130, in-frame deletion mutants of both PA4130 and PA4129 were constructed to generate strains PAJD25 and PASF06, respectively. These isogenic mutants showed no growth differences in either lysogeny broth (LB) or artificial sputum medium (ASM) in relation to the parental strain (see Fig. S2 in the supplemental material).

10.1128/mBio.00207-21.1 TABLE S1 Twitching, swimming, protease, and elastase production in PA4130 interrupted strains. Download Table S1, DOCX file, 0.1 MB. Copyright © 2021 Fenn et al. 2021 Fenn et al. https://creativecommons.org/licenses/by/4.0/ This content is distributed under the terms of the Creative Commons Attribution 4.0 International license.

10.1128/mBio.00207-21.5 FIG S1 Growth of PAO1-L, PAJD25, and PASF06 in LB and modified artificial sputum medium. Download FIG S1, PDF file, 0.1 MB. Copyright © 2021 Fenn et al. 2021 Fenn et al. https://creativecommons.org/licenses/by/4.0/ This content is distributed under the terms of the Creative Commons Attribution 4.0 International license.

10.1128/mBio.00207-21.6 FIG S2 Amino acid alignment performed with ClustalW comparing PA4130 with homologous proteins CysI (E. coli), NirA (spinach), and SirA (M. tuberculosis). Download FIG S2, PDF file, 0.1 MB. Copyright © 2021 Fenn et al. 2021 Fenn et al. https://creativecommons.org/licenses/by/4.0/ This content is distributed under the terms of the Creative Commons Attribution 4.0 International license.

The PAJD25 strain showed phenotypes similar to those of the Tn*5* mutant PAJD21, with pyocyanin and pyoverdine reduced in both LB and ASM and swarming motility also impeded. Chromosomal complementation of PAJD25 with PA4130 restored pyocyanin and swarming motility to wild-type levels, with partial restoration of pyoverdine production (Fig. 1A to D). In contrast, the PA4129 deletion mutant PASF06 did not show any significant alterations in the virulence-related phenotypes (Table S3). This demonstrates that the phenotypes observed are specific to the mutation of PA4130.

10.1128/mBio.00207-21.3 TABLE S3 Virulence factor production by strains PASF06 and PAJD25 compared to the isogenic wild-type strain. Download Table S3, DOCX file, 0.04 MB. Copyright © 2021 Fenn et al. 2021 Fenn et al. https://creativecommons.org/licenses/by/4.0/ This content is distributed under the terms of the Creative Commons Attribution 4.0 International license.

**FIG 2:**
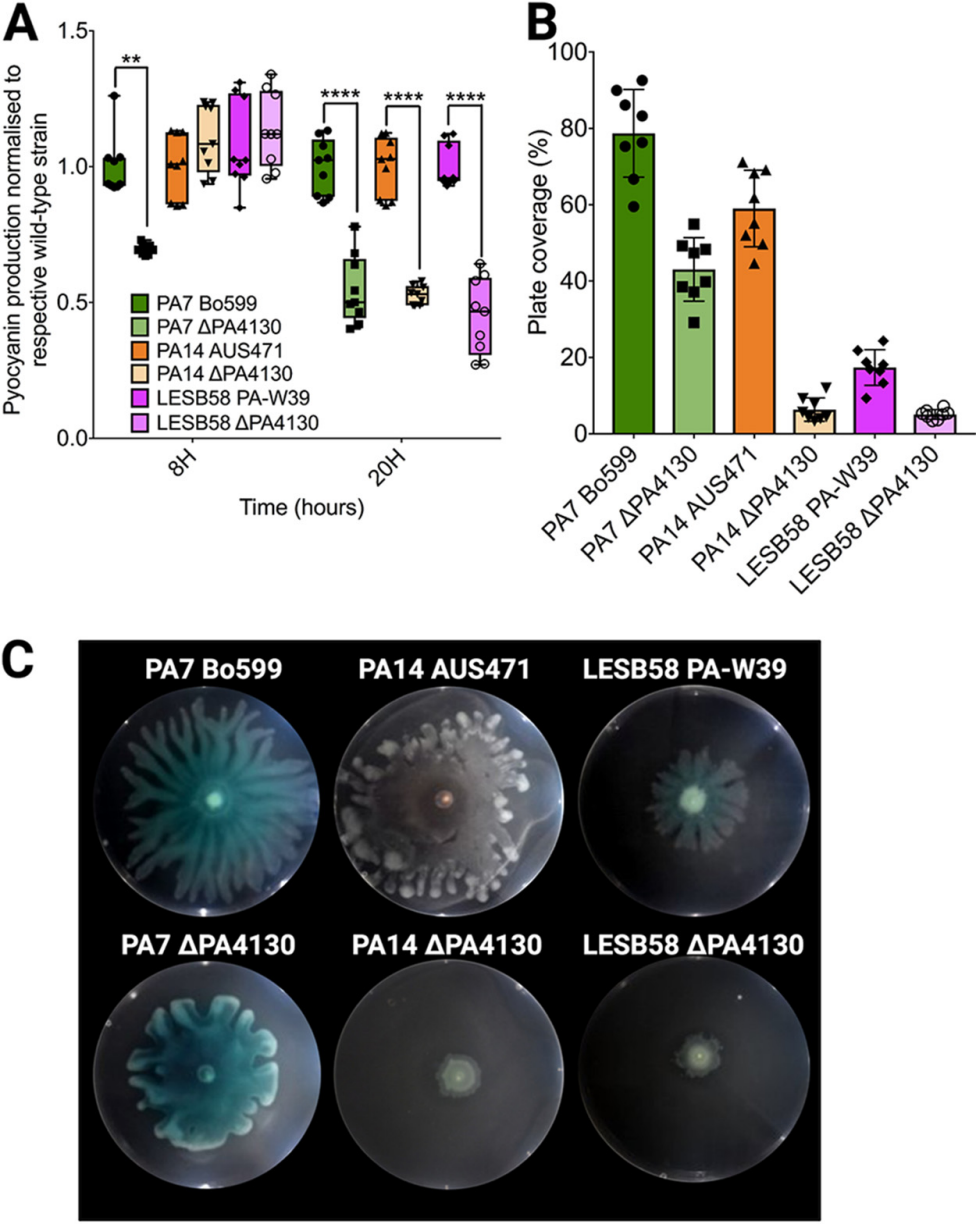
*In vitro* phenotypic characterization of virulence factor production for PA4130 orthologue mutants in PA7 Bo599, PA14 AUS471, and LESB58 PA-W39. (A) Pyocyanin levels determined in supernatants from cultures grown for 8 and 20 h in LB using a colorimetric assay. (B and C) Swarming motility surface coverage measurements (B) and images (C); cells were inoculated from a 16-h LB culture onto swarming plates containing 0.5% agar and incubated at 37°C for 20 h. Data were collated from two independent experiments with at least four replicates. Values that are significantly different by Tukey’s multiple-comparison test are indicated by asterisks as follows: *, *P* < 0.05, **, *P* < 0.01, ****, *P* < 0.0001.

When identifying new virulence targets, it is paramount to ensure they are conserved in a wide range of strains. To establish this, a nucleotide BLAST search of PA4130 against the NCBI P. aeruginosa taxonomic identifier database (taxid 287) revealed that this hypothetical protein is highly conserved, with 363/367 of the available genomes encoding a PA4130 orthologue. To determine whether PA4130 has a similar role in the virulence of P. aeruginosa strains from different sublines, in-frame PA4130 deletion mutants were created in the clinical isolates PA7 Bo599, PA14 AUS471, and LESB58 PA-W39. Deletion of PA4130 across all these strains resulted in a similar attenuation for both pyocyanin production and swarming motility, while protease production and growth remained unaffected, validating the results obtained for PAJD21 and PAJD25 (Fig. 2A to C and Table S4). In contrast, reduction in pyoverdine production was not conserved with no consistent pattern emerging between strains (Table S4). The similar phenotypes observed in the PA4130 deletion strains across phylogenetically diverse P. aeruginosa strains confirms PA4130 is not a PAO1-L specific virulence determinant. This supported the use of PAO1-L and the derived strain PAJD25 as representative strains to further characterize the role of PA4130 in virulence.

10.1128/mBio.00207-21.4 TABLE S4 Virulence factor production from PA4130 orthologue mutant strains compared to the isogenic wild-type strain. Download Table S4, DOCX file, 0.05 MB. Copyright © 2021 Fenn et al. 2021 Fenn et al. https://creativecommons.org/licenses/by/4.0/ This content is distributed under the terms of the Creative Commons Attribution 4.0 International license.

### PA4130 is required for full virulence in C. elegans and D. melanogaster.

**FIG 3:**
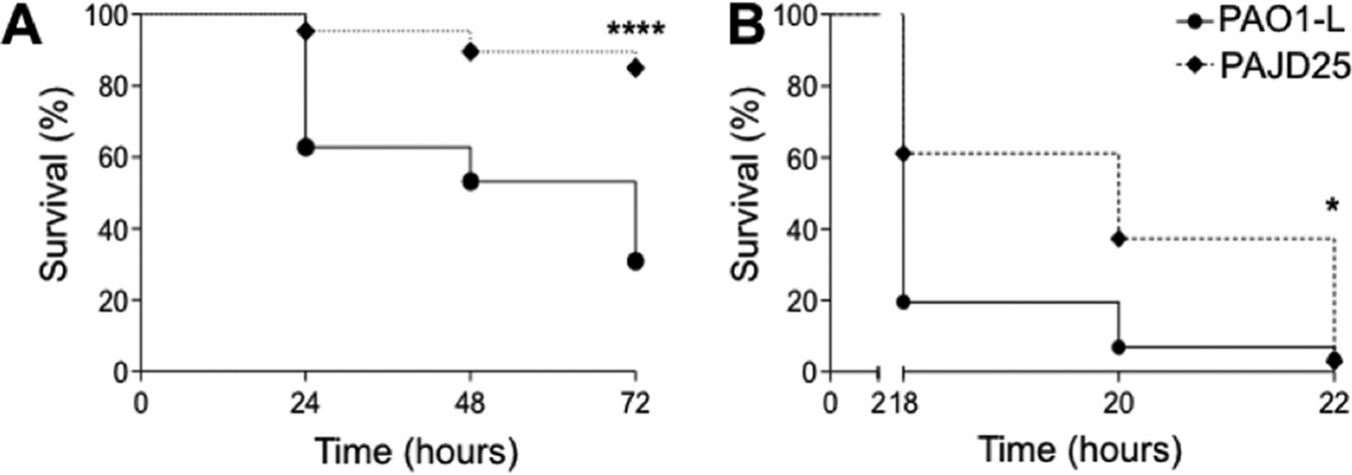
Survival of strain PAO1-L versus strain PAJD25 in nonmammalian models. (A) 72-h lethality curve in the C. elegans infection model. (B) 22-h lethality curve in D. melanogaster. Both models demonstrated clear attenuation with increased survival of PAJD25 in C. elegans at all time points. D. melanogaster exhibited a delay in killing with increased survival at earlier time points; however, by 22 h, both PAO1-L and PAJD25 showed almost complete killing. The results presented display the mean values from three independent experiments. *, *P* < 0.032, ****, *P* < 0.0001 log rank Mantel-Cox test.

To establish whether the decrease in virulence trait production observed in the PA4130 deletion mutant PAJD25 *in vitro* also results in disease attenuation *in vivo*, the D. melanogaster and C. elegans nonmammalian infection models previously used for P. aeruginosa infection studies were initially used. In both disease models, the PAJD25 (ΔPA4130) mutant showed an attenuated survival rate compared to that of the isogenic wild-type PAO1-L (Fig. 3). For C. elegans, PAJD25 virulence was severely attenuated with a 57% increase in survival at 72 h postinfection (Fig. 3A). In the case of D. melanogaster, PAJD25 killing was delayed with a 40% increase in survival at 18 h postinfection, although by 22 h, this difference was negligible (Fig. 3A).

### Deletion of PA4130 results in reduced cytotoxicity, cellular invasion, and IL-8 production in A549 human lung epithelial cell line.

**FIG 4:**
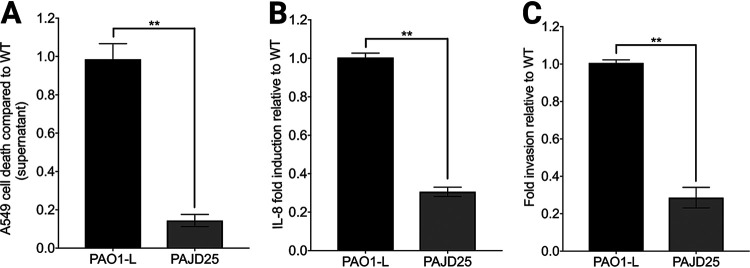
Cytotoxicity, IL-8 release, and invasion of A549 cells following infection with either strain PAO1-L or PAJD25. (A) Cytotoxicity assayed with Syto13/propidium iodine (PI) viability staining. WT, wild type. (B) IL-8 released quantified by enzyme-linked immunosorbent assay (ELISA) following infection. (C) Invasion quantified using an antibiotic (polymyxin B) exclusion assay. Data from three independent experiments are expressed as means ± standard errors of means (SEM) (error bars). **, *P* < 0.01 by Student’s *t* test.

A previous screening of the Tn*5* mutant library screening by Dubern and colleagues (23) suggested that attenuation using *in vitro* assays and invertebrate infection models does not translate into mammalian models. To establish whether this is the case for the PA4130 mutant, the PAJD25 (ΔPA4130) strain was tested on the A549 pulmonary cell line for cytotoxicity, invasion, and interleukin 8 (IL-8) production (Fig. 4). Cytotoxicity of the PAJD25 supernatant was drastically reduced compared to strain PAO1-L, which is in line with the reduced levels of secreted pyocyanin (Fig. 1A and 4A). Furthermore, invasion of A549 epithelial cells was reduced in strain PAJD25, with IL-8 production also reduced compared to wild-type strain PAO1-L (Fig. 4B and C).

### Deletion of PA4130 reduces lethality of P. aeruginosa in acute and agar bead murine infection models.

**FIG 5:**
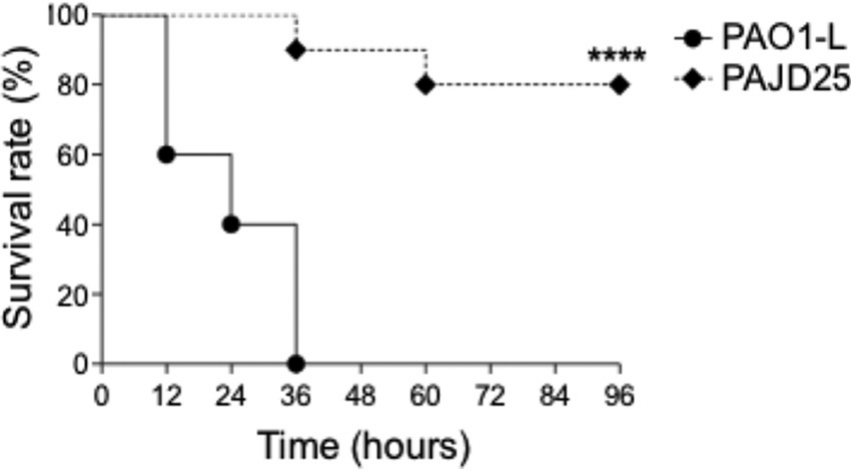
Survival curve of C57BL/6NCrlBR mice infected with strains PAO1-L and PAJD25 in an acute lung infection model. At 36 h, PAJD25 exhibits a 90% increase in survival compared to PAO1-L, with 80% of PAJD25-infected mice surviving past 96 h. ****, *P* < 0.0001 by log rank Mantel-Cox test.

The impact of a PA4130 mutation on P. aeruginosa virulence was initially assessed by determining the survival curve of PJD25 (ΔPA4130) in an acute lung infection model in C57BL/6NCrlBR mice using an infection dose of 5 × 10^6^ CFU. A survival rate of 80% was shown at 72 h postinfection in contrast to strain PAO1-L which did not show any survival after 36 h (Fig. 5).

**FIG 6:**
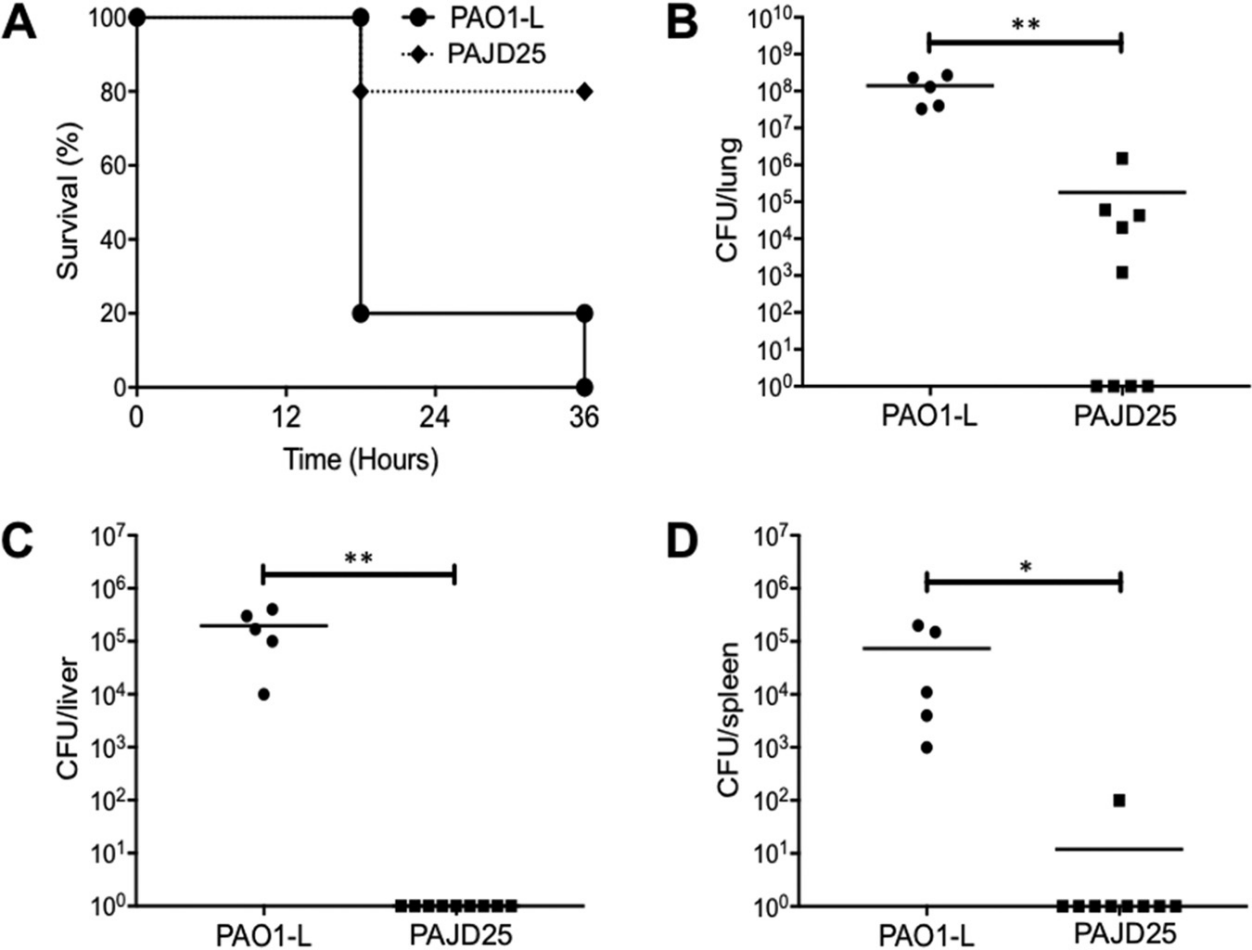
Virulence of strains PAO1-L and PAJD25 in an agar bead murine lung infection model of colonization in C57BL/6NCrlBR mice. (A to D) Survival curve (A), CFU recovery from lung (B), CFU recovery from the liver (C) and CFU recovery from the spleen at 36 h postinfection (D). Strain PAJD25 exhibits a significant increase in survival at 36 h postinoculation with a CFU recovery 3 log units lower than that of strain PAO1-L in the lungs. PAO1-L infection progresses to the liver and spleen within 36 h, while PAJD25 infection does not proceed to the liver and spleen as indicated by the absence of CFU recovery in all but one sample. ****, *P* < 0.0001 by log rank Mantel-Cox test for survival curve. *, *P* < 0.05; **, *P* < 0.01 by Student’s *t* test for CFU recovery.

The effect of the PA4130 mutation was then tested in an agar bead infection model to monitor initial colonization and systemic spread. C57BL/6NCrlBR mice were infected with a 2 × 10^6^ CFU dose of either strain PAO1-L or PAJD25. At 18 h postinfection, 20% of PAO1-L-infected mice and 80% of PAJD25-infected mice survive. By 36 h, 80% of PAJD25-challenged mice still survive while PAO1-L-infected mice exhibit no survival (Fig. 6A). The reduction in mortality was confirmed by the reduced CFU recovery from the lung, liver, and spleen of the mutant compared to the wild type (Fig. 6B to D). Clearance or reduced CFU in the lung of PAJD25-infected mice demonstrates that interruption of PA4130 interferes with colonization. This impaired colonization results in reduced systemic spread with no PAJD25 detected in the spleen or liver in all but one sample (Fig. 6B to D).

Overall, the data presented demonstrate that PA4130 plays a role in P. aeruginosa survival and virulence in multiple models of infection, suggesting that PA4130 is not a model-specific virulence gene.

### Purification of PA4130 reveals characteristics of a possible nitrite or sulfite reductase.

Amino acid sequence analysis showed that PA4130 has an amino acid similarity of 21% with the Escherichia coli CysI sulfite reductase hemoprotein subunit and that no protein homologues are encoded within the human genome. Alignment of the PA4130 amino acid sequence with the E. coli MG1655 CysI, Mycobacterium tuberculosis H37RV NirA, and Spinacea oleracea (spinach) NirA, using ClustalW, revealed the presence of conserved residues indicative of 4Fe-4S and siroheme prosthetic group binding sites (Fig. S2). Initial expression and purification attempts yielded insoluble, nonfunctional PA4130. Prosthetic group limitation was hypothesized to limit soluble PA4130 expression. Cooverexpression of iron-sulfur cluster biogenesis or siroheme synthesis proteins has been demonstrated to increase expression of functional enzymes incorporating these prosthetic groups (24, 25). PA4130 was subsequently cooverexpressed in the presence of the siroheme synthase CysG, yielding a 20-fold increase in soluble PA4130 expression. PA4130 was purified to homogeneity with a combination of immobilized metal affinity chromatography and chitin column chromatography, while size exclusion chromatography revealed PA4130 is a monomer in solution (Fig. S3). Spectroscopic characterization of the soluble PA4130 protein confirmed the presence of peaks at 382 nm, 587 nm, and 712 nm, characteristic of siroheme and 4Fe-4S incorporation into nitrite/sulfite reductase hemoprotein subunits (data not shown).

10.1128/mBio.00207-21.7 FIG S3 PA4130 purification using E. coli NiCo21 pSK4130-N/pCDF-*cysG*. Download FIG S3, PDF file, 0.1 MB. Copyright © 2021 Fenn et al. 2021 Fenn et al. https://creativecommons.org/licenses/by/4.0/ This content is distributed under the terms of the Creative Commons Attribution 4.0 International license.

### PA4130 codes for an assimilatory nitrite reductase operating in a ferredoxin-dependent manner.

Nitrite or sulfite reductases catalyze the six-electron reduction of nitrite or sulfite to ammonium or hydrogen sulfide, respectively. These enzymes require an electron acceptor and donor to complete the electron transport chain. The electron acceptor is nitrite or sulfite with the electron donor being either NADPH, NADH, or ferredoxin.

**FIG 7:**
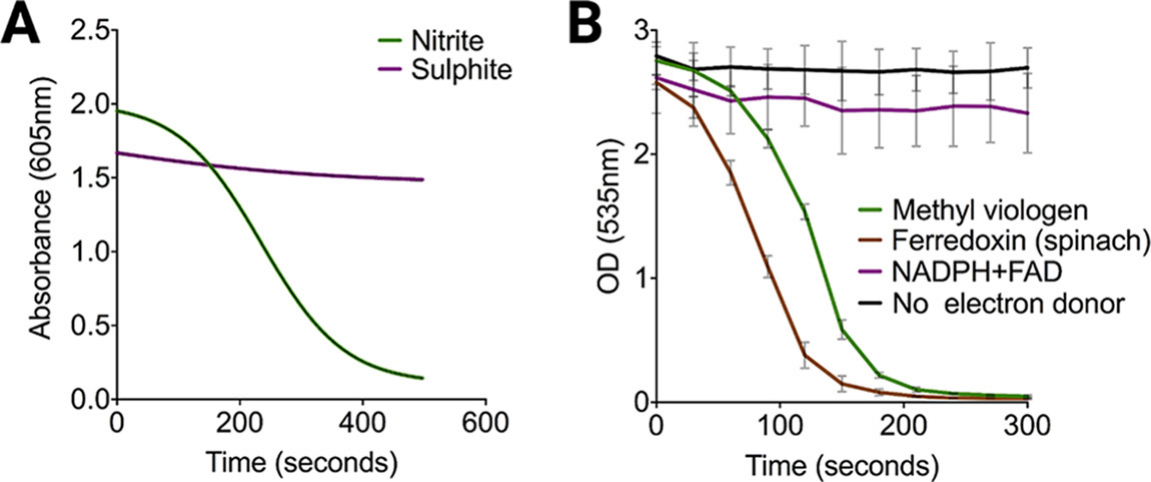
PA4130-mediated reduction assays for native electron acceptor and donor determination. (A) Methyl viologen oxidation assay in the presence of the electron acceptors nitrite and sulfite. Reduced MV is oxidized only in the presence of nitrite, indicating PA4130 is a nitrite reductase. (B) Nitrite reduction assay using MV, reduced ferredoxin, and NADPH as electron donors. The remaining nitrite was quantified by Griess diazotization, with nitrite reduction occurring in the presence of MV and reduced ferredoxin.

To determine the native electron acceptor, we used a reduced methyl viologen (MV) reductase assay, which spectrophotometrically tracks the artificial electron donor oxidation in the presence of nitrite or sulfite as the possible electron acceptor for PA4130 (26). Our data show that PA4130 was able to oxidize MV only in the presence of nitrite with minimal oxidation occurring in the presence of sulfite (Fig. 7A). These results confirm that PA4130 functions as a nitrite reductase and hence participates in the nitrate reduction pathway.

To identify the native electron donor used by PA4130, the same assay as described above was carried out with the alternative electron donors NADPH+FAD and reduced spinach ferredoxin, with the remaining nitrite quantified using Griess diazotization. Tracking of the remaining nitrite revealed that reduction occurs only in the presence of MV and reduced ferredoxin while minimal reduction occurs with NADPH plus FAD (Fig. 7B). This observation is in agreement with a study by Frangipani and colleagues showing that increased expression of both PA4130 and the ferredoxin:NADP^+^ reductase *fprA* are induced by cyanogenesis in P. aeruginosa (27). This suggests that the role of FprA is to reduce ferredoxins, using NADPH as an electron donor, ensuring a supply of reduced ferredoxin for PA4130 to derive electrons for nitrite reduction.

**FIG 8:**
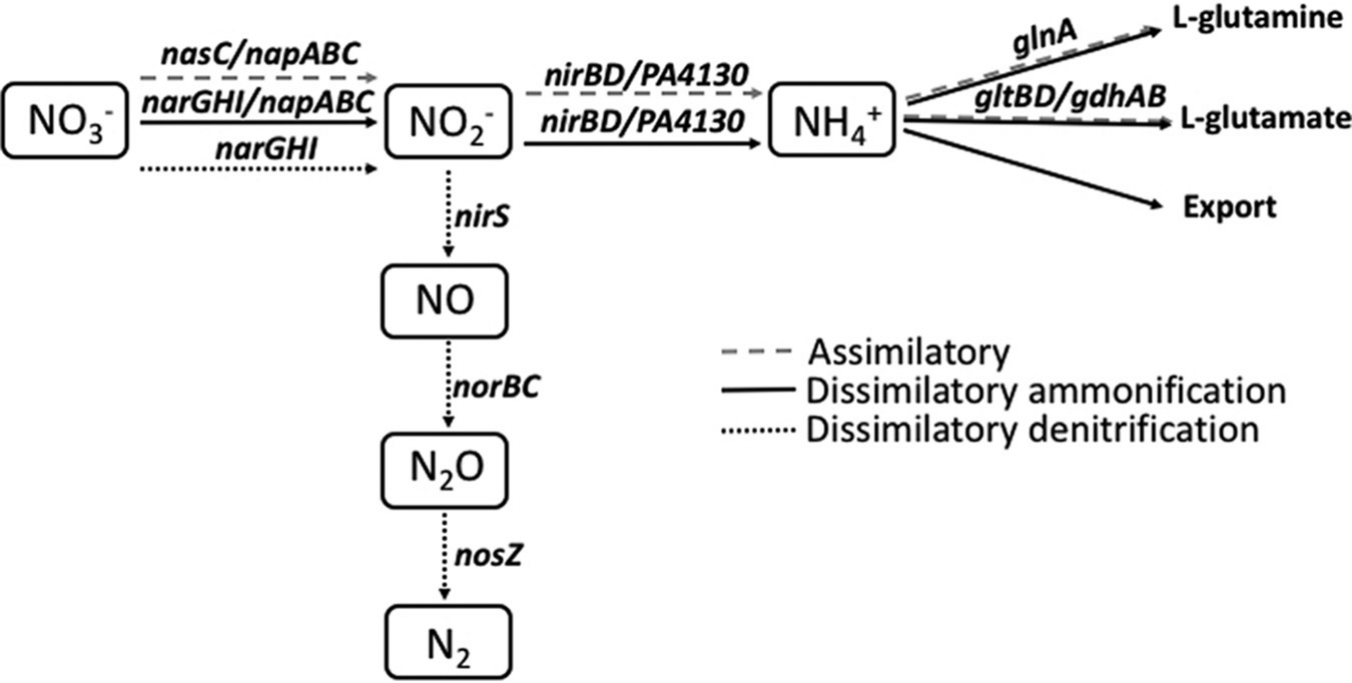
Genetic and biochemical pathways involved in nitrate reduction. This highly branched process is divided into three primary pathways: assimilatory and dissimilatory nitrite reduction to ammonium (DNRA) and dissimilatory denitrification. The presence of multiple pathways enables multiple biological functions to be performed with amino acid synthesis, detoxification, energy generation, and conservation key to survival in diverse environments.

The nitrite reductase cognate pathways can be assigned via the end reaction product. Nitric oxide (NO)-forming nitrite reductases participate in the dissimilatory denitrification pathway, while ammonium (NH_4_^+^)-producing reductases are part of either assimilatory or dissimilatory nitrite reduction to ammonium (DNRA) pathways (Fig. 8) (28). P. aeruginosa genes encode three structurally distinct nitrite reductases, the NO-forming denitrification reductase NirS, NH_4_^+^-forming NirBD, and unassigned PA4130. Conserved domain analysis using DELTA-BLAST suggests that PA4130 resembles an ammonium-producing nitrite reductase. This was confirmed with an ammonium production assay following nitrite reduction using reduced ferredoxin as an artificial electron donor (Fig. S4).

10.1128/mBio.00207-21.8 FIG S4 Detection of remaining nitrite and ammonium production in a reduced ferredoxin-dependent nitrite reduction assay with PA4130. Download FIG S4, PDF file, 0.01 MB. Copyright © 2021 Fenn et al. 2021 Fenn et al. https://creativecommons.org/licenses/by/4.0/ This content is distributed under the terms of the Creative Commons Attribution 4.0 International license.

Together, these data indicate that PA4130 codes for an ammonium-producing, ferredoxin- and siroheme-dependent, nitrite reductase, which participates in the nitrate reduction cycle. We propose that PA4130 be renamed NirA to fit with current nomenclature.

## DISCUSSION

In a previous study, we aimed to identify novel genes required for virulence in P. aeruginosa using a multihost screening strategy with the ultimate goal of discovering novel antivirulence targets (23). Using the same strategy we have identified, a new Tn*5* mutation in PA4130 which is attenuated in multiple virulence factor production. One limitation previously identified with this screening strategy (23) was the use of a single strain of P. aeruginosa, as there can be significant variations between the virulence of different strains, potentially leading to the identification of strain-specific virulence factors (29). This problem was circumvented by generating PA4130 orthologue deletion mutants in phylogenetically distinct P. aeruginosa strains covering the major phylogenetic groups identified by Freschi and colleagues (30). This revealed a conserved role of PA4130 in virulence factor production with pyocyanin production and swarming motility being impaired across all mutants. Further screening revealed attenuation of a PA4130 mutant across all disease models tested in this study, confirming that PA4130 is not a disease model-specific virulence determinant and is likely to play a key role in survival during human infection. This makes PA4130 a promising target which can be taken forward for functional and structural characterization with a view to develop novel virulence inhibitors against this opportunistic pathogen.

Characterization of the predicted gene product of PA4130 showed an assimilatory ferredoxin-dependent nitrite reductase likely involved in nitrate and wider central nitrogen metabolism. Hence, it has subsequently been named *nirA*. Previous studies have also detected various carbon and nitrogen metabolic genes as essential for full virulence, since infection is associated with significant metabolic changes (23, 31, 32). Inhibiting adaptation to carbon and nitrogen sources or attenuating the ability to protect from nitrative stress during infection could prove an attractive antivirulence strategy with metabolic dysregulation having knockdown effects on multiple cellular cycles.

Nitrogen source metabolism is essential for the survival of P. aeruginosa in diverse environments enabling amino acid synthesis, carbon source utilization, and respiration in the absence of oxygen (31). A key element to nitrogen source metabolism is the reduction of nitrate. P. aeruginosa nitrate metabolism is a highly branched and interlinked process with three main pathways, dissimilatory denitrification, dissimilatory nitrate reduction to ammonium (DNRA), and assimilatory nitrate reduction to ammonium (Fig. 8) (28, 33).

Dissimilatory denitrification in P. aeruginosa reduces nitrate to gaseous dinitrogen, coupling reduction to generation of a proton gradient. This facilitates respiration in the absence of oxygen. The assimilatory pathway is dedicated to biosynthesis, with the assimilatory nitrate reductase (NasC) and assimilatory nitrite reductase (NirBD) producing nitrogen in the form of ammonium. DNRA differs, requiring the respiratory nitrate reductases NarGHI or NapABC to enable energy generation/conservation (28, 33) while simultaneously maintaining intracellular level of nitrogen via the production of ammonium.

The NirA production of ammonium *in vitro* suggests that it participates in either assimilatory or DNRA with potential implications in amino acid biosynthesis, energy conservation, detoxification, and maintenance of the intracellular redox environment (28, 34). However, determining the exact role NirA plays during virulence is complicated by the fact that P. aeruginosa genes code for two additional producing nitrite reductases, nitric oxide-forming NirS and ammonium-forming NirBD.

NirS is a component of the dissimilatory denitrification pathway and reduces nitrite to nitric oxide (NO). Various components of the dissimilatory nitrate reduction pathway have been demonstrated to be attenuated for virulence, with deletion of NirS in PA14 reported to be avirulent in C. elegans and the THP-1 human monocyte cell line (35, 36). However, these NirS-dependent phenotypes have been attributed to production of NO which modulates type III secretion system expression (36). Since NirA is an NH_4_-forming nitrite reductase, it participates in a separate part of the nitrate reductase pathway (Fig. 8), with the resulting phenotypes independent of NO production.

Although NirBD and NirA perform the same molecular function, they are differentially regulated. *nirBD-PA1779*(*nasC*)*-cobA* are under the control of the RpoN nitrogen utilization sigma factor, with NtrC and NasT acting as transcriptional activators. Expression occurs under low nitrogen availability in the presence of nitrate and nitrite (37). In contrast, NirA expression is under the control of cyanogenesis and is involved in protecting cells from HCN self-intoxication alongside PA4129-34 and *cioAB* by a yet undefined mechanism (27).

The role played by these ammonium-forming nitrite reductases during pathogenesis has largely remained unexplored *in vivo* with the exception of M. tuberculosis, where induction of *nirBD* is required for survival in human macrophages during hypoxia (38). Animals and plants produce reactive nitrogen species (RNS) in response to diverse bacterial infections (39). Host-derived RNS originate from inducible and constitutively expressed nitric oxide synthases (iNOS), with subsequent autooxidation resulting in formation of nitrate and nitrite (40). P. aeruginosa is capable of detoxifying these RNS and actively uses them for respiration and macromolecule biosynthesis, thriving under challenging conditions.

With induction of *nirA* under the control of HCN, maximal expression will occur under microaerobic conditions due to the dual action of Anr and the *las*/*rhl* quorum-sensing cascade (41). This suggests that NirA plays a similar role to NirBD of M. tuberculosis, allowing persistence or growth *in vivo* by protecting P. aeruginosa from nitrative stress under oxygen limiting conditions. Work is now ongoing to understand the role of NirA in virulence and how this overlaps with the function of NirBD, NirS, and cyanogenesis.

While the mechanisms are yet to be unraveled, the discovery that the nitrite reductase NirA is required for virulence in multiple infection models represents a very attractive prospect for the development of inhibitors. Humans do not reduce nitrate and nitrite and as such do not encode nitrite reductases, minimizing off-site effects of any inhibitors developed. Taking these points together makes NirA a promising antivirulence target candidate against P. aeruginosa infections.

## MATERIALS AND METHODS

### Bacterial strains, plasmids, growth conditions, and DNA manipulation.

Bacterial strains and plasmids are listed in Table S1 in the supplemental material. P. aeruginosa and E. coli were routinely cultured in lysogeny broth (LB) at 37°C with vigorous shaking (200 rpm), unless otherwise stated. Artificial sputum medium (ASM) was made according to reference 42, modified with the addition of 0.5 mM KNO_3_ to replicate conditions reported in the cystic fibrosis (CF) lung (43, 44). Media were solidified with 1.5% agar. Antibiotics were added, when required, at concentrations of 20 μg ml^−1^ for gentamicin, 15 μg ml^−1^ for nalidixic acid, and 20 μg ml^−1^for streptomycin. Tetracycline was added at a final concentration of 150 μg ml^−1^ for P. aeruginosa and 10 μg ml^−1^ for E. coli. Protein overexpression was performed in terrific broth (TB) (24 g/liter yeast extract, 12 g/liter tryptone, 4% glycerol, 0.017 M KH_2_PO_4_, and 0.072 K_2_HPO_4_) supplemented with 1 mM FeSO_4_·6H_2_O and 50 μg ml^−1^ of carbenicillin and streptomycin.

Genomic DNA isolation was performed using a Wizard Genomic DNA purification kit (Promega). Plasmid isolation was performed with GenElute plasmid miniprep kit (Sigma). All other standard DNA manipulation techniques such as analysis, digestion, ligation, and transformation were performed according to Sambrook and Russell (45).

### Mutant selection and phenotype confirmation.

A previously reported P. aeruginosa Tn*5* mutant library was further screened for new mutants showing alterations in pyocyanin production, swarming motility, and alkaline protease activity with the aid of Flexis (Genomics solutions) colony-picking robot as previously described (23). Quantitative assays confirming observed phenotypes for pyocyanin, pyoverdine, swarming, and protease were evaluated as described elsewhere (46).

### C. elegans and D. melanogaster virulence assays.

Both nematode slow killing assays and D. melanogaster disease models were performed as previously described (23, 47).

### Cell culture, IL-8 secretion, invasion, and cytotoxicity assays.

A549 (human type II pneumocytes) were purchased from ATCC and cultured as described previously (48). IL-8 secretion was performed as reported previously (23). Bacterial invasion was determined using a polymyxin B protection assay (23). Cytotoxicity of P. aeruginosa culture supernatant and cell pellet was assessed using the Syto-13/propidium iodide viability test according to reference 49.

### In-frame deletion mutant construction and complementation.

To construct an in-frame deletion in PA4130, two DNA fragments 427 bp upstream and 433 bp downstream from PA4130 were generated and fused by overlap extension PCR using PAO1-L genomic DNA as a template. The upstream 427-bp fragment was amplified with primers PA4130F1 which carries an XbaI restriction site and PA4130R1 containing the first 12 nucleotides of PA4130 with an overhanging end containing the last 15 nucleotides of the PA4130 ORF (Table S1); the downstream 433-bp fragment was amplified with PA4130F2 containing the last 15 nucleotides of PA4130 with an overhanging end containing the first 12 nucleotides and PA4130R2 containing a HindIII restriction site (Table S1). To perform the overlap extension PCR, a secondary PCR was performed with the 427-bp and 433-bp fragments serving as the templates and primers PA4130F1/PA4130R2 (Table S2). The final PCR product was cloned using the XbaI/HindIII restriction sites into the vector pME3087, resulting in the suicide plasmid pME4130. The suicide plasmid used to generate the PA4129 mutant was constructed as described above, using primer pairs PA4129F1/PA4129R1 and PA4129F2/PA4129R2 to generate two PCR products upstream and downstream of PA4129 and using primer pairs PA4129F1/PA4129R2 to generate the final PCR product containing a deletion in PA4129, which was cloned into pME3087, resulting in the suicide plasmid pME4129 (Table S2).

10.1128/mBio.00207-21.2 TABLE S2 Bacterial strains, plasmids, and primers used in this study. Download Table S2, DOCX file, 0.1 MB. Copyright © 2021 Fenn et al. 2021 Fenn et al. https://creativecommons.org/licenses/by/4.0/ This content is distributed under the terms of the Creative Commons Attribution 4.0 International license.

The PA4129 and PA4130 in-frame deletions were generated by allelic exchange using pME4129 and pME4130, respectively. Briefly, pME4129 and pME4130 were mobilized by conjugation into the relevant P. aeruginosa strains using E. coli S17.1 λ*pir*. Conjugants were selected for on LB agar supplemented with tetracycline and nalidixic acid. Strains were restreaked twice on LB, with no antibiotic, and subjected to tetracycline sensitivity enrichment to select for double crossover events (49). Colonies were screened for loss of resistance to tetracycline with allelic exchange confirmed with PCR and DNA sequencing, resulting in strains PASF06 (ΔPA4129), PAJD25 (PAO1-L ΔPA4130), PA7 Bo599 ΔPA4130, PA14 AUS471 ΔPA4130, and LESB58 ΔPA4130 (Table S2).

Strain PAJD25 was complemented by amplifying a 2,172-bp fragment containing the PA4130 open reading frame (ORF) and +498 bp of the translational start site using primers 4130CTXF1/4130CTXR1. The PCR product was cloned into the integrative vector mini-CTX-1 using the HindIII/BamHI restriction sites, forming pCTX4130, with the resulting vector mobilized by conjugation, as performed with pME4129 and pME4130. Conjugants were selected on LB agar supplemented with tetracycline and nalidixic acid. The integration of pCTX4130 was confirmed using PCR and DNA sequencing with complementation demonstrated through restoration of pyocyanin production and swarming motility.

### Acute murine infection model.

C57BL/6NCrlBR male mice (8 to 10 weeks of age) were purchased from Charles River Laboratories, Italy. In the acute murine lung infection model, P. aeruginosa strains were grown for 3 h in tryptic soy broth (TSB). Bacteria were then harvested, washed twice with sterile phosphate-buffered saline (PBS), and resuspended in sterile PBS to the desired dose for infection of 5 × 10^6^ CFU/mouse. Mice were anaesthetized, and the trachea were directly visualized by a ventral midline incision, exposed, and intubated with a sterile, flexible 22-g cannula attached to a 1-ml syringe accordingly to established procedures (48). A 60-μl inoculum of 5 × 10^6^ CFU was implanted into the lung via cannula. Following infection, mice were monitored twice a day for 4 days. Mice that lost >20% body weight and presented signs of severe clinical disease were sacrificed by CO_2_ administration before termination of the experiment. Further details are outlined in supplemental material.

### Agar bead infection model.

C57BL/6 male mice (6 to 10 weeks of age) were purchased from Charles River Laboratories, Germany. The agar bead mouse model was performed according to established procedures (50). Fresh cultures were prepared in 5 ml TSB and incubated for 3 h. Bacterial cells were harvested and embedded in agar beads according to Bragonzi and colleagues (51). Five to 10 mice were used for experiments and intratracheally infected with 4.6 × 10^6^ CFU. Following infection, mice were monitored twice a day for 2 days. Mice that lost >20% body weight and presented signs of severe clinical disease were sacrificed by injection of 2 ml of 20% pentobarbital. For quantitative bacteriology, lung, liver, and spleen were excised aseptically and homogenized using the homogenizer DIAX 900 (Heidolph GmbH, Schwabach, Germany). Bacterial numbers in the organs were determined by 10-fold serial dilutions of the homogenates, spotted onto blood plates after incubation at 37°C for 18 h.

### Ethics statement.

Acute murine infection studies were conducted according to protocols approved by the San Raffaele Scientific Institute (Milan, Italy) Institutional Animal Care and Use Committee (IACUC) and adhered strictly to the Italian Ministry of Health guidelines for the use and care of experimental animals. Agar bead infection studies were conducted according to protocols approved by Institute of Medical Microbiology and Hygiene (Tübingen, Germany) and adhered strictly to guidelines set by the German Ministry of Health and Animal Welfare Institute (Baden-Württemberg).

### PA4130 expression and purification.

The PA4130 *orf* was amplified from PAO1 genomic DNA using primer pair NT4130F1/NT4130R1 containing N-terminal hexahistidyl tag and EcoRI/SacI restriction sites (Table S2). The modified PA4130 fragment was cloned into vector pSK67 using EcoRI/SacI restriction sites, resulting in plasmid pSK4130-N. The *cysG orf* was PCR amplified from E. coli BL21(DE3) using primer pair CDFcysGF1/CDFcysGR1 containing restriction sites BglII/XhoI (Table S2). The modified *cysG* gene was inserted into vector PCDF-DUET1 using restriction sites BglII/XhoI, producing vector pCDF-cysG. The resulting pSK4130-N and pCDF-*cysG* coding sequences were confirmed with commercial Sanger sequencing.

Vectors pSK4130-N and pCDF-*cysG* were cotransformed into E. coli NiCo21 by electroporation with simultaneous selection of both vectors on LB agar plates using carbenicillin and streptomycin. Single colonies were selected and grown in LB broth for 16 h at 37°C. Cell density was then adjusted to an optical density (OD) of 0.05 into TB and further grown to an OD of ∼0.6 to 0.8 (600 nm). Cultures were then cooled to 20°C before addition of 0.05 M ferric citrate and induction with 0.1 mM isopropyl-β-d-thiogalactopyranoside. Following a further 18-h growth, cell pellets were harvested via centrifugation, flash frozen in liquid N_2_, and stored at −80°C.

For purification, a combination of immobilized metal ion affinity chromatography (IMAC), chitin column chromatography (CCC) used to obtain soluble PA4130. Frozen pellets were resuspended in lysis buffer consisting of 50 mM Tris-HCl, 150 mM NaCl, 1.2 μg/ml lysozyme, and cOmplete Ultra EDTA-free protease inhibitor cocktail at pH 8.0 with 1 g cell paste per 10 ml of buffer. Samples were incubated on ice for ∼30 min with membrane disruption achieved by sonication (Fisher model 505/705).

IMAC was performed with the HiTrap chelating HP column (GE Healthcare) charged with NiSO_4_. The column was equilibrated with 10 column volumes (CV) of buffer A containing 50 mM Tris-HCl, 500 mM NaCl, 5% glycerol, 20 mM imidazole at pH 8.0 before sample application with a peristaltic pump (GE Healthcare model P1). PA4130 was obtained using a linear imidazole gradient from 20 to 400 mM imidazole between buffer A and buffer B (buffer A with 400 mM imidazole) with red/brown samples collected and pooled together.

Following IMAC, samples were processed and applied to chitin resin according to the manufacturer’s instructions (New England Biolabs). The CCC flowthrough harboring PA4130 was collected and analyzed by sodium dodecyl sulfate-polyacrylamide gel electrophoresis (SDS-PAGE) for homogeneity. For size-exclusion chromatography, samples were concentrated to 5 ml using a Vivaspin centrifugal filter unit (Cytiva) and injected onto a 16/600 Superdex 200-pg column (Cytiva) and eluted with 50 mM Tris and 150 mM NaCl (pH 7.5).

### Reduced methyl viologen assay.

The PA4130 protein was tested for nitrite/sulfite reductase activity using the artificial electron donor methyl viologen (MV); the MV reduction reaction in the presence of sodium dithionite produces a blue coloration. The assay was adapted from an assay of Schnell and colleagues (25) under anaerobic conditions to prevent oxygen-dependent oxidation of MV. MV oxidation was tracked spectrophotometrically at 5-s intervals with nitrite, hydroxylamine, or sulfite acting as an electron acceptor.

### Griess diazotization and ammonia detection assays.

Griess diazotization was performed using the Griess reagent system (Promega) according to manufacturer’s instructions. The assays were performed as described above, with the exception that the electron donors were altered. MV, spinach ferredoxin, and NADPH plus flavin mononucleotide (FMN) were used, with MV and ferredoxin artificially reduced using sodium dithionite. Temporal tracking of reduction reaction was achieved via sacrifice of eight independent reactions every 30 s.

Production of ammonia was confirmed using an ammonia assay kit (Sigma) according to the manufacturer’s instructions.
