# Supplementary material for: NirA Is an Alternative Nitrite Reductase from Pseudomonas aeruginosa with Potential as an Antivirulence Target
Source: mBio. 2021 Apr 20;12(2):e00207-21. doi: 10.1128/mBio.00207-21 (PMC8092218; doi:10.1128/mBio.00207-21)
Supplement: TABLE S1 [file mBio.00207-21-st001.docx]

**Table S1** Twitching, swimming, protease and elastase production in PA4130 interrupted strains.
