# Supplementary material for: NirA Is an Alternative Nitrite Reductase from Pseudomonas aeruginosa with Potential as an Antivirulence Target
Source: mBio. 2021 Apr 20;12(2):e00207-21. doi: 10.1128/mBio.00207-21 (PMC8092218; doi:10.1128/mBio.00207-21)

FIG S1 Growth curves of PAO1-L, PAJD25 (ΔPA4130) and PASF06 (ΔPA4129) in (A) LB and (B) Modified artificial sputum media demonstrating no growth defect of the mutants when compared to WT.

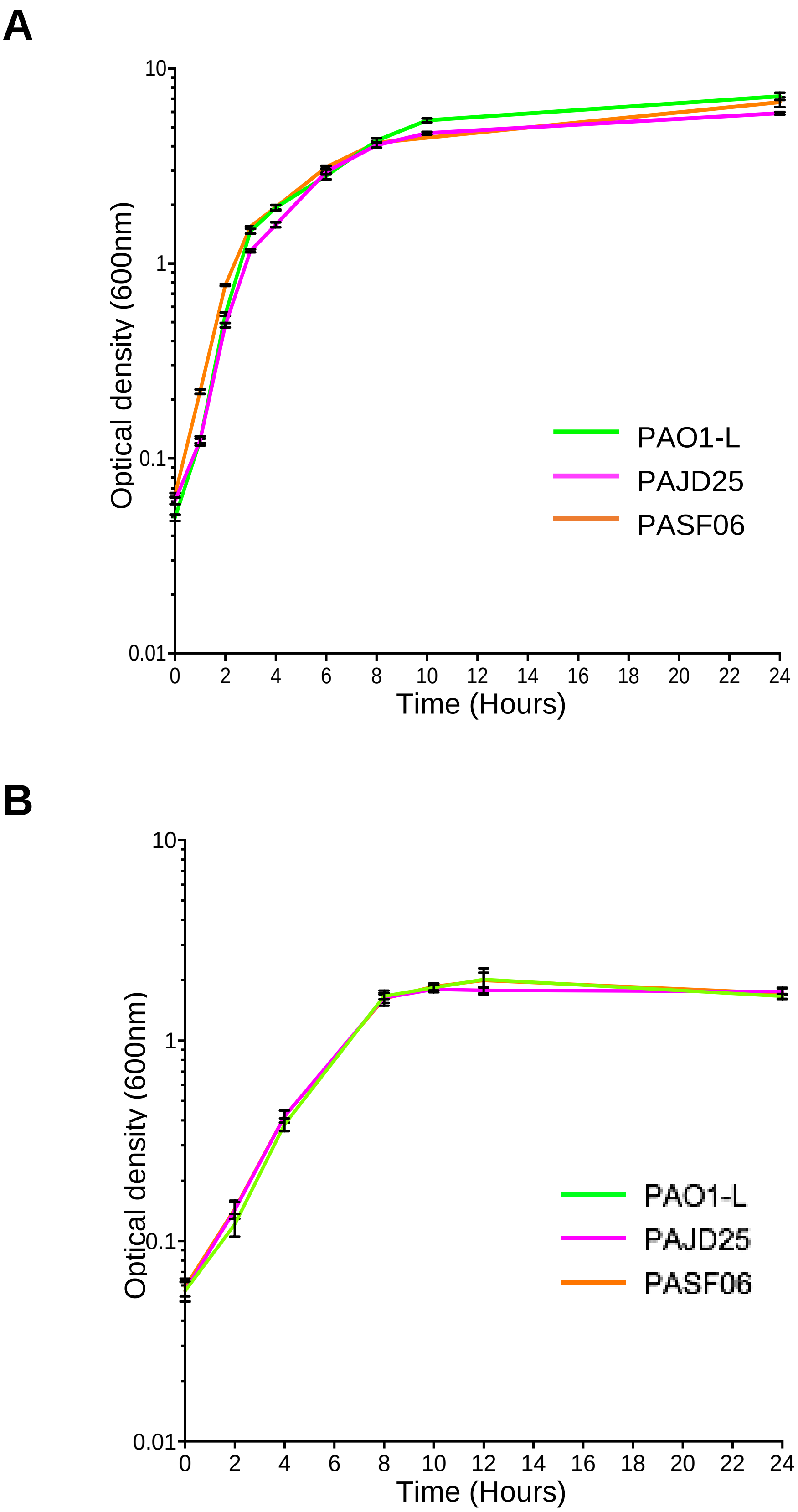

Supplement: FIG S1 [file mBio.00207-21-sf001.pdf]
