# Supplementary material for: NirA Is an Alternative Nitrite Reductase from Pseudomonas aeruginosa with Potential as an Antivirulence Target
Source: mBio. 2021 Apr 20;12(2):e00207-21. doi: 10.1128/mBio.00207-21 (PMC8092218; doi:10.1128/mBio.00207-21)
Supplement: FIG S2 [file mBio.00207-21-sf002.pdf]

FIG S2 Amino Acid alignment performed with ClustalW comparing PA4130 with homologous proteins CysI (*E. coli*), NirA (Spinach) and SirA (*M. tuberculosis*). Highlighted in green are Siroheme interacting residues observed in the crystal structures of CysI, NirA and SirA with conserved residues of PA4130 highlighted in pink. Conservation of these residues along with four essential cysteine residues required for iron-sulfur cluster insertion suggests that PA4130 requires Siroheme and 4Fe-4S for its functional activity.

```
EcoliCysI      ---------------------------------------------------------MSE
SpinachNirA    MASLPVNKIIPSSTTLLSSSNNNRRRNNSSIRCQKAVSPAAETAAVSPSVDAARLEPRVE
PA4130         -------------------------------------------------------MYQYD
M_tubSirA      ---------------------------------------------------MTTARPAKA

EcoliCysI      KHPGPLVV----EGKLTDAERMKHESNYLRGTIAEDLNDGLTGGFKGDNFLLIRFH----
SpinachNirA    ERDGFWVLKEEFRSGINPAEKVKIEKDPMKLFIEDGISDLAT--LSMEEVDKSK-HNKDD
PA4130         EYDQALV------------SERVAQFRD--------QIARRLDGELSEEEFLPLRLQN---
M_tubSirA      RNEGQWAL--GHREPLNANEELKKAGNPL------DVRERIENIYAKQGFDSI---DKTD

EcoliCysI      --------GMYQQDDRDIR----AERAEQKLEPRHAMLLRCRLPGGVITTKQWQAIDKFA
SpinachNirA    IDVRLKWLGLFHRRKHHYG----R------------FMMRLKLPNGVTTSEQTRYLASVI
PA4130         --------GLYLQKHA--------------------YMLRVAIPYGTLSAPQLRALAHVA
M_tubSirA      LRGRFRWWGLYTQREQGYDGTWTGDDNIDKLEAKY-FMMRVRCDGGALSAAALRTLGQIS

EcoliCysI      GENTIYGSIRLTNRQTFQFHGILKKNVKPVHQMLHSVGLDALATANDMNRNVLCTSNPYE
SpinachNirA    KKYGKDGCADVTTRQNWQIRGVVLPDVPEIIKGLESVGLTSLQSGMDNVRNPV--GNPLA
PA4130         RHYDR-GYGHFTTRQNIQFNWIELEQVGDILEHLAGAQMHAIQTSGNCVRNIT--TEAFA
M_tubSirA      TEFAR-DTADISDRQNVQYHWIEVENVPEIWRRLDDVGLQTTEACGDCPRVVL--GSPLA

EcoliCysI      SQLHAEAYEWAKKISEHLLPRTR-AYAEIWLDQEKVATTDEEPILGQTYLPRKFKTTVVI
SpinachNirA    GI---DPHEIVD---------TR-PFTNLI---SQFVTANSRGNLSITNLPRKWNPCVIG
PA4130         GV---AADEWTD---------PR-PLAEIL---RQWSTVNP----EFLFLPRKFKIALSS
M_tubSirA      GE---SLDEVLD---------PTWAIEEIV---RRYIG-KP----DFADLPRKYKTAISG

EcoliCysI      PPQNDIDLHANDMNFVAI--AENGKLVGFNLLVGGGLSIEHGNKKTYARTASEFGYLPLE
SpinachNirA    SHDLYEHPHINDLAYMPA--TKNGKF-GFNLLVGGFFSIKRCEEAIPL-----DAWVSAE
PA4130         AVEDRAAVQMHDIGLYLYRHPDAGEL-RLRVLVGGGLG------RTPMLGQVIRDDLPWQ
M_tubSirA      LQD--VAHEINDVAFIGVNHPEHGP--GLDLWVGGGLS------TNPMLAQRVGAWVPLG

EcoliCysI      HTLAVAEAVVTTQRDWGNRTDRKNAKTKYTLERVGVETFKAEVERRAGIKFEPIRPY---
SpinachNirA    DVVPVCKAMLEAFRDLGFRGNRQKCRMMWLIDELGMEAFRGEVEKRMPEQVLERASS---
PA4130         HLLSYVEAILRVYNRYGRRDNKYKARIKILVKALGIEAFAREVEEEW--QHLRDGPAQLT
M_tubSirA      EVPEVWAAVTSVFRDYGYRRLRAKARLKFLIKDWGIAKFREVLETEYLKRPLIDGPA---

EcolicysI      ----------------------------------EFTG----RGDRIGWVKGIDDNWHLT
SpinachNirA    ---------------------------------EELVQKDWERREYLGVHPQKQQGLSFV
PA4130         AEECQRVAERFVLPRYLPPADGELAYGSARAADPAFAR--WASR-NVQAHK-VPGYASVV
M_tubSirA      ---------------------------------PEPVK---HPIDHVGVQR-LKNGLNAV

EcolicysI      LFIENGRILDYPARPLKTGLLE-----IAKIHKGDFRITANQNLIIAGVPESEKAKIEKI
SpinachNirA    -----G--LHIPVGRLQADEMEELARIADVYGSGELRLTVEQNIII---PNVENSKIDSL
PA4130         LSTKPG--ASAPPGDVTAEQMERVADWAERYGFGEIRVAHEQNLVL---PDVRLENLHAL
M_tubSirA      -----G--VAPIAGRVSGTILTAVADLMARAGSDRIRFTPYQKLVILDIPDALLDDLIAG

EcolicysI      AKESGLMNAVTP----QRENSMACVSFPTCPLA----MAEAERFLPSFIDNIDNLMAKHG
SpinachNirA    LNEPLLKERYSPEPPILMKGLVACTGSQFCGQAIIETKARALKVTEEVQ-RLVSVTR---
PA4130         WREACAAGLGTPNQG-LLSDIIACPGGDYCALA----NAKSIPIAQGIQQRFEDLDHLHD
M_tubSirA      LDALGLQSRPSH----WRRNLMACSGIEFCKLSFAETRVRAQHLVPELERRLEDINSQLD
```

```
EcolicysI      VSDEHIVMRVTGCPNGCGRAMLAEVGLVG----KAPGR----YNLHLGGNR-IGTRIPRM
SpinachNirA    --P--VRMHWTGCPNSCGQVQVADIGFMGCMTRDENGKPCEGADVFVGGRIGSDSHLGDI
PA4130         IGE--LSLNISGCMNACGHHHIGNIGILGV---DKSGS--EWYQVTLGGAQGKDSALGKV
M_tubSirA      V-P--ITVNINGCPNSCARIQIADIGFKGQMIDDGHGGSVEGFQVHLGGHLGLDAGFGRK

EcolicysI      YKE-NITEPEILASLDELIGRW---AKEREAGEGFGDFTVRAGI--IRPVLDPARDLWD
SpinachNirA    YKKAVPCKDLVPVVAEILINQFGAVPREREEAE--------------------------
PA4130         IGP-SFSAAEVPAVIERIVETF---TDLRVGPERFIDTFNRVGLEPFKARVYARMEEPA
M_tubSirA      LRQHKVTSDELGDYIDRVVRNF---VKHRSEGERFAQWVIRAEEDDLR-----------
```
