# Supplementary material for: NirA Is an Alternative Nitrite Reductase from Pseudomonas aeruginosa with Potential as an Antivirulence Target
Source: mBio. 2021 Apr 20;12(2):e00207-21. doi: 10.1128/mBio.00207-21 (PMC8092218; doi:10.1128/mBio.00207-21)
Supplement: TABLE S3 [file mBio.00207-21-st003.docx]

**Table S3** Virulence factor production from strains PASF06 (ΔPA4129) and PAJD25 (ΔPA4130) expressed as a percentage (%) of the wild type strain PAO1-L. Data collected from at least 2 separate experiments with 3 to 5 replicates.

| **Assay** | **PASF06** | **PAJD25** |
| --- | --- | --- |
|  |  |  |
| Pyocyanin | 88±10 | 48±7** |
| Pyoverdine | 94±12 | 67±10* |
| Swarming surface coverage | 106±7 | 20±4**** |
| Elastase | 91±7 | 108±5 |
| Protease | 90±9 | 92±5 |
