# Supplementary material for: NirA Is an Alternative Nitrite Reductase from Pseudomonas aeruginosa with Potential as an Antivirulence Target
Source: mBio. 2021 Apr 20;12(2):e00207-21. doi: 10.1128/mBio.00207-21 (PMC8092218; doi:10.1128/mBio.00207-21)
Supplement: TABLE S4 [file mBio.00207-21-st004.docx]

**Table S4** Virulence factor production from PA4130 orthologue mutant strains expressed as a percentage (%) of the wild-type strain at 20H. Data collated from 2 separate experiments with 3 to 5 replicates.

| **Assay** | **PA7 Bo599 PA4130** | **PA14 AUS471 PA4130** | **LESB58 PA-W39 PA4130** |
| --- | --- | --- | --- |
| Pyocyanin | 54.7±10**** | 52.7±3.5**** | 44.9±9.3**** |
| Pyoverdine | 140±6**** | 53.7±2.5 | 206±31**** |
| Swarming surface coverage | 56.2±9.2**** | 12.7±2.5**** | 42.3±8.5* |
| Protease | 110±12.9 | 89.7±9.1 | 111.1±6.6 |
