# Supplementary material for: NirA Is an Alternative Nitrite Reductase from Pseudomonas aeruginosa with Potential as an Antivirulence Target
Source: mBio. 2021 Apr 20;12(2):e00207-21. doi: 10.1128/mBio.00207-21 (PMC8092218; doi:10.1128/mBio.00207-21)

Fig S3 PA4130 purification using *E. coli* NiCo21 pSK4130-N/pCDF-*cysG*.(A) Protein purification was performed with a combination of immobilised metal ion chromatography (IMAC) and chitin column chromatography (CCC). Lane 1- broad-range MWt ladder; lane 2- Soluble lysate fraction; lane 3- lysate IMAC flow-through; lane 4- IMAC 40mM imidazole wash; lane 5- post IMAC PA4130 sample; lane 6- CCC flow-through; lane 7-CCC wash.(B) Size exclusion chromatogram displaying elution profile of PA4130 post-NINTA. PA4130 flows as a monomer with a predicted molecular weight of 61kDa.

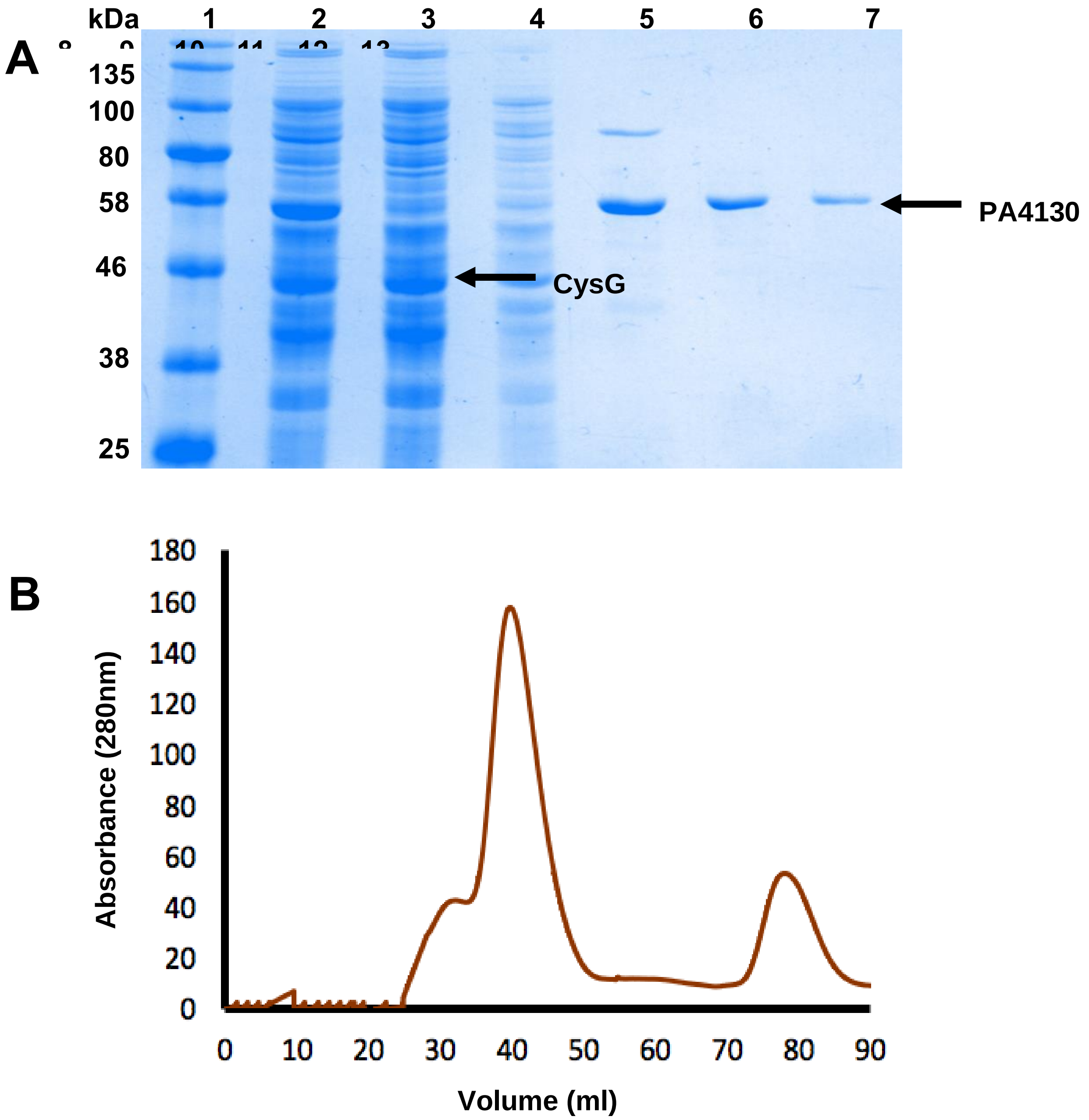

Supplement: FIG S3 [file mBio.00207-21-sf003.pdf]
