# Supplementary material for: NirA Is an Alternative Nitrite Reductase from Pseudomonas aeruginosa with Potential as an Antivirulence Target
Source: mBio. 2021 Apr 20;12(2):e00207-21. doi: 10.1128/mBio.00207-21 (PMC8092218; doi:10.1128/mBio.00207-21)

Fig S4 Detection of remaining nitrite and ammonium production in a reduced ferredoxin dependant nitrite reduction assay with PA4130. Nitrite levels decrease concomitantly with an increase in ammonium production at a 1:1 ratio. Nitrite and ammonium concentrations were determined using Griess diazotisation and an ammonia assay kit. Concentrations were calculated through interpolation of absorbance values on standard curves prepared with KNO_2 and NH_4Cl over a range of 0-120umol.

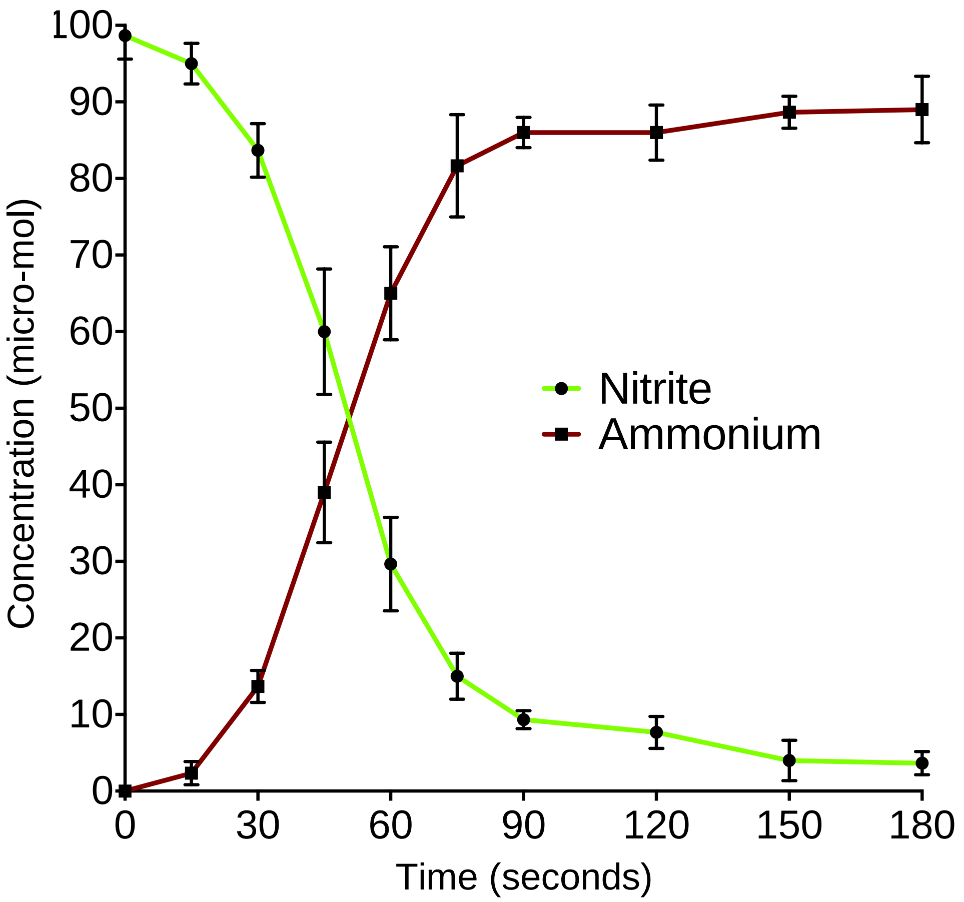

Supplement: FIG S4 [file mBio.00207-21-sf004.pdf]
