# Supplementary material for: NirA Is an Alternative Nitrite Reductase from Pseudomonas aeruginosa with Potential as an Antivirulence Target
Source: mBio. 2021 Apr 20;12(2):e00207-21. doi: 10.1128/mBio.00207-21 (PMC8092218; doi:10.1128/mBio.00207-21)
Supplement: TABLE S2 [file mBio.00207-21-st002.docx]

**Table S2** Bacterial strains, plasmids and primers used in this study.

| **Strains** | **Description** | **Origin** |
| --- | --- | --- |
| ***P. aeruginosa* strains** |  |  |
|  |  |  |
| PAO1-L | PAO1 Lausanne collection wild type | (1, 2) |
| PAJD21 | Strain PAO1-L with Tn5 insertion in PA4130 Gm^R^ | This study |
| PASF06 | In-frame marker-less deletion of PA4129 | This study |
| PAJD25 | In frame marker-less deletion of PA4130 | This study |
| PA7 Bo599 | Clinical PA7 strain | (3) |
| PA7 Bo599 ΔPA4130 | In-frame marker-less deletion of PA4130 orthologue | This study |
| PA14 AUS471 | Clinical PA14 strain | (3) |
| PA14 AUS471 ΔPA4130 | In-frame marker-less deletion of PA4130 orthologue | This study |
| LESB58 PA-W39 | Clinical LESB58 strain isolated from wound. | (3) |
| LESB58 PA-W39 ΔPA4130 | In-frame marker-less deletion of PA4130 orthologue | This study |
|  |  |  |
| ***E. coli* strains** |  |  |
|  |  |  |
| NEB5-alpha | F−,φ80dl*acZ*ΔM15,Δ(*lacZYA-argF*)U169,*deoR*,*recA1*, *endA1*, *hsdR17*(rk−,mk+), *phoA*, *supE44*, λ−, thi1, *gyrA96*, *relA1* | New England Biolabs |
| S17.1 λpir | pro, res^−^ *hsdR17* (rK^−^ mK^+^) *recA^−^* with an integrated RP4-2-Tc::Mu-Km::Tn7, Tp^r^ *λpir* | (4) |
| BL21 (DE3) | F– *ompT* *gal* *dcm lon* *hsdSB*(rB–mB–) λ(DE3 [*lacI* lacUV5-T7p07 *ind1* *sam7* *nin5*]) [*malB+*]K-12(λS) | (5) |
| NiCo21 (DE3) | F– *ompT* *gal dcm lon hsdSB*(rB–mB–) λ(DE3 [*lacI* lacUV5-T7p07 *ind1* *sam7* *nin5*]) [*malB+*]K-12(λS) *glmS6ala* *slyD-CBD* *arnA-CBD* | (6) |
|  |  |  |
| **Plasmids** |  |  |
|  |  |  |
| pME3087 | Suicide vector, ColE1 replicon, Tc^R^ | (7) |
| pME4129 | pME3087 based vector with upstream and downstream regions of PA4129 spliced together for markerless deletion generation. | This study |
| pME4130 | pME3087 based vector with upstream and downstream regions of PA4130 spliced together for double-crossover generation | This study |
| Mini-CTX-1 | *aatB* P. aeruginosa integrative vector, Tc^R^ | (8) |
| pCTX4130 | Integrative PA4130 complementation vector under control of the native promoter (+498bp), Tc^R^ | This study |
| pSK67 | pTOPO type vector for protein overexpression. Under control of a T7 promoter and IPTG inducible. Amp^R^ |  |
| pSK4130 | pSK67 based vector with N-terminal hexahistidyl tagged PA4130 inserted at the EcoR1 and SacI restriction sites for overexpression | This study |
| pCDF-DUET1 | DUET vector containing 2 T7 promoters under the control of LacI. Enables co-expression of up to 4 target proteins. Sp^R^ | Novagen |
| pCDF-*cysG* | pCDF-DUET1 based vectors with *cysG* inserted into MCS2 at the NcoI and XhoI site for overexpresssion | This study |
|  |  |  |
| **Primers** | **Sequence** | **Modifications** |
| 4129DELF1 | 5’-ATAGAATTCTGTGGCGCGAGGCCTGCG-3’ | EcoRI |
| 4129DELR1 | 5’-CTAGCGTCGGCGGAACAGGTTGTTCATGCCGGTTCC-3’ | N/A |
| 4129DELF2 | 5’-GGCATGAACAACCTGTTCCGCCGACGCTAGGCATAC-3’ | N/A |
| 4129DELR2 | 5’-TATGGATCCGCTGGAACAGCGTGGCGGAG-3’ | BamHI |
| 4130DELF1 | 5’-ATATCTAGATCATTTTTCGTAGGCCCATC-3’ | XbaI |
| 4130DELR1 | 5’-TCATGCCGGTTCCTCGTACTGGTACATCGCAAAGCC-3’ | N/A |
| 4130DELF2 | 5’-GCGATGTACCAGTACGAGGAACCGGCATGAACAACC-3’ | N/A |
| 4130DELR2 | 5’-ATAAAGCTTTCCTCGACGTTCTTGTCCTC-3’ | HindIII |
| 4130CTXF1 | 5’-ATAAAGCTTGGGCCGTTCACCGCCGAC-3’ | HindIII |
| 4130CTXR1 | 5’-TATGGATCCTCATGCCGGTTCCTCCATCCTG-3’ | BamHI |
| NTH-30F1 | 5’TTAGAATTCATGCATCACCATCACCATCACTACCAGTACGATGAATACG-3’ | EcoRI + 6XHis |
| NTH-30R1 | 5’-TTACTCGAGTCATGCCGGTTCCTCCATCCT-3’ | SacI |
| M2cysGF1 | 5’-ATACCATGGGTGGATCATTTGCCTATATTTTGC-3’ | NcoI |
| M2cysGR1 | 5’-TATGGATCCTTAATGGTTGGAGAACCAGTTCAG-3’ | XhoI |

**Supplemental references**

1. Lyon BR, Skurray R. 1987. Antimicrobial resistance of *Staphylococcus aureus*: genetic basis. Microbiol Rev 51:88-134.

2. Heurlier K, Denervaud V, Pessi G, Reimmann C, Haas D. 2003. Negative control of quorum sensing by RpoN (sigma54) in *Pseudomonas aeruginosa* PAO1. J Bacteriol 185:2227-35.

3. Freschi L, Jeukens J, Kukavica-Ibrulj I, Boyle B, Dupont MJ, Laroche J, Larose S, Maaroufi H, Fothergill JL, Moore M, Winsor GL, Aaron SD, Barbeau J, Bell SC, Burns JL, Camara M, Cantin A, Charette SJ, Dewar K, Deziel E, Grimwood K, Hancock RE, Harrison JJ, Heeb S, Jelsbak L, Jia B, Kenna DT, Kidd TJ, Klockgether J, Lam JS, Lamont IL, Lewenza S, Loman N, Malouin F, Manos J, McArthur AG, McKeown J, Milot J, Naghra H, Nguyen D, Pereira SK, Perron GG, Pirnay JP, Rainey PB, Rousseau S, Santos PM, Stephenson A, Taylor V, Turton JF, Waglechner N, et al. 2015. Clinical utilization of genomics data produced by the international *Pseudomonas aeruginosa* consortium. Front Microbiol 6:1036.

4. Metcalf WW, Jiang W, Wanner BL. 1994. Use of the rep technique for allele replacement to construct new *Escherichia coli* hosts for maintenance of R6K gamma origin plasmids at different copy numbers. Gene 138:1-7.

5. Studier FW, Moffatt BA. 1986. Use of bacteriophage T7 RNA polymerase to direct selective high-level expression of cloned genes. J Mol Biol 189:113-30.

6. Robichon C, Luo J, Causey TB, Benner JS, Samuelson JC. 2011. Engineering Escherichia coli BL21(DE3) derivative strains to minimize *E. coli* protein contamination after purification by immobilized metal affinity chromatography. Appl Environ Microbiol 77:4634-46.

7. Voisard C, Bull CT, Keel C, Laville J, Maurhofer M, Schnider U, Défago G, Haas D. 1994. Biocontrol of root diseases by *Pseudomonas fluorescens* CHA0: current concepts and experimental approaches. Molecular ecology of rhizosphere microorganisms 73:2504-2514.

8. Hoang TT, Kutchma AJ, Becher A, Schweizer HP. 2000. Integration-proficient plasmids for *Pseudomonas aeruginosa*: site-specific integration and use for engineering of reporter and expression strains. Plasmid 43:59-72.
